# Controlled-Release Fertilizers: Innovations, Challenges, and Practical Applications

**DOI:** 10.3390/molecules31172979

**Published:** 2026-08-26

**Authors:** Mariusz Siudak, Maciej Combrzyński, Tomasz Oniszczuk, Jakub Soja, Anna Oniszczuk

**Affiliations:** 1 Department of Food Process Engineering, University of Life Sciences in Lublin, Głęboka Street 31, 20-612 Lublin, Poland; siudak.mariusz@gmail.com (M.S.); maciej.combrzynski@up.edu.pl (M.C.); jakub.soja@up.edu.pl (J.S.); 2 Department of Inorganic Chemistry, Medical University of Lublin, Chodźki 4a, 20-093 Lublin, Poland

**Keywords:** controlled-release, crop yield, environmental effects, sustainable agriculture, soil health, agriculture, extrusion

## Abstract

Ensuring high crop productivity while reducing nutrient losses and environmental impacts remains a central challenge of modern fertilization strategies. Controlled-release fertilizers (CRFs) have emerged as a key tool to synchronize nutrient availability with plant demand by embedding soluble nutrient sources within coatings or matrices that modulate water penetration and ion diffusion. This narrative review synthesizes recent advances in CRF materials and technologies, with particular emphasis on biodegradable and bio-based systems, hydrogels, nanostructured carriers, and extrusion-based matrix formulations. It outlines the main classes of coating and matrix materials, their release mechanisms, agronomic performance, and documented benefits for nutrient-use efficiency, crop yield and quality, and soil and water protection. The review also analyzes major scientific, technical, environmental, and economic barriers that currently limit the large-scale deployment of CRFs, including microplastic pollution from persistent polymer coatings and the difficulty of tailoring release profiles under variable field conditions. Emerging directions are highlighted, such as composite and multilayer bio-based coatings, superhydrophobic and stimuli-responsive structures, biochar- and compost-based matrices, nutrient recovery from waste streams, and integration with precision agriculture and sensor technologies. The paper identifies priorities for future research needed to translate promising CRF concepts into robust, field-validated and sustainable fertilization solutions.

## 1. Introduction

A major focus of contemporary nutrient management research is the challenge of sustaining high agricultural productivity while simultaneously mitigating environmental impacts. Food systems around the world are currently operating under mounting pressure resulting from population growth, progressive soil degradation, intensifying climate stresses, and the need to increase fertilizer use efficiency without exacerbating negative impacts on soil, water, and atmospheric ecosystems. In this context, fertilizer technologies cannot be evaluated solely in terms of their ability to intensify crop yields, but also in terms of their potential to synchronize nutrient supply with the dynamics of plant demand, which directly determines the reduction in nutrient losses throughout the entire production cycle [1,2]. Conventional mineral fertilizers remain the primary tool for intensifying crop production, as they provide nutrients in chemically defined forms that are highly soluble and readily available to plants. At the same time, the high solubility of these fertilizers makes them susceptible to leaching, surface runoff, volatilization, immobilization, and other loss mechanisms that reduce nutrient use efficiency and exacerbate the environmental impacts of their use. Thus, plants utilize only a portion of the applied nutrients, while a significant proportion of nitrogen, phosphorus, and potassium is lost to the environment, contributing to water eutrophication, soil acidification, greenhouse gas emissions, groundwater contamination, and long-term soil quality degradation [3,4]. Consequently, improving nutrient use efficiency is one of the key priorities in both scientific research and agronomic practice. A classic method of reducing losses is split fertilization, which allows for better alignment of nutrient supply with the successive stages of plant development; however, this approach involves increased labor, greater sensitivity to weather conditions, and the risk of errors related to the timing and location of application [5,6]. These limitations have contributed to the rapid development of more efficient fertilizers, among which controlled-release fertilizers (CRFs) are currently considered one of the most promising strategies for regulating nutrient availability in the soil environment [7]. CRFs are defined as fertilizer formulations containing water-soluble nutrient sources whose release into the soil environment is intentionally delayed and modulated by coatings, encapsulating barriers, or matrix systems with precisely engineered physicochemical properties. CRFs understood in this way belong to a group of fertilizers with enhanced efficacy, in which the material architecture (type and thickness of the coating, porosity, hydrophobicity, and degree of matrix cross-linking) is directly linked to the kinetic profile of nutrient release and its alignment with the dynamics of plant nutrient demand over time [4,8].

The agronomic rationale for using CRFs is highly compelling. By slowing the rate at which nutrients are released and extending the period during which they remain available in the soil solution, CRFs can significantly reduce nutrient losses. In many cases, this translates not only into higher yields but also into improved crop quality, resulting from a more balanced supply of macro- and micronutrients to plants [7]. In addition, CRFs can provide environmental benefits, such as improved soil water retention, reduced ammonia volatilization, reduced nitrate leaching into groundwater, and increased efficiency in the spatial distribution of nutrients, particularly when these fertilizers are combined with biochar, hydrogels, or a targeted application strategy focused on the rhizosphere [9]. Despite these advantages, CRFs are not a solution free of technological and environmental limitations. Their widespread use continues to be hindered by relatively high production costs, resulting, among other things, from the need for multi-stage coating processes and the use of advanced polymer materials, as well as by the difficulty of precisely tailoring the nutrient release profile to diverse soil and climatic conditions and to the specific needs of crop species and varieties under field conditions. Another significant issue is environmental concerns related to conventional coating materials, particularly petrochemical-based polymers, which, after the nutrient release phase has ended, may persist in the soil as poorly biodegradable residues, contributing to plastic accumulation and the potential degradation of soil ecosystem functions [10].

Although many publications describe new materials or specific formulations, there remains a need for a synthesis that links the development of CRFs to current challenges in soil fertilization. Accordingly, this narrative review analyzes the development of CRF fertilizers, the principles governing nutrient release, the materials and manufacturing strategies used in their production, as well as the major scientific, technical, environmental, and economic constraints that continue to hinder their wider adoption. It also highlights emerging solutions and future directions, with a particular focus on biodegradable materials and advanced formulation strategies.

The literature search was conducted using the PubMed, Scopus, Web of Science and Google Scholar databases. The following queries were used: “inorganic fertilizers”, “coated fertilizers”, “biopolymers”, “organic fertilizers”, “slow-release fertilizers”, “controlled-release fertilizers”, “sustainable agriculture”, “soil health”, “bio-based materials”, “field cultivation”. Only papers published after January 2008 were included. To qualify for inclusion in the review, papers had to meet the following criteria: contain original data; have been independently reviewed; be written in English; and have been published after January 2008.

## 2. Classification of Controlled-Release Fertilizers

Controlled-release fertilizers are products containing water-soluble nutrients whose release into the soil is intentionally delayed or regulated by an insoluble barrier, carrier, matrix, or coating, so that nutrient availability is spread out over time, rather than occurring immediately after application. They differ significantly from conventional soluble fertilizers due to their ability to more closely match nutrient supply to plant demand, which improves nutrient use efficiency and reduces losses resulting from leaching, runoff, volatilization, and other related environmental processes. Enhanced-efficiency fertilizers comprise several product categories that should not be considered interchangeable. In particular, slow-release fertilizers (SRFs) and controlled-release fertilizers (CRFs) may both delay nutrient availability relative to conventional soluble fertilizers, but they differ in the origin and controllability of the release process. A consistent classification must therefore distinguish nutrient-release mechanism, formulation architecture, material composition, manufacturing route, and release-performance criteria [4,10].

### 2.1. Slow-Release Versus Controlled-Release Fertilizers

**Figure 1:**
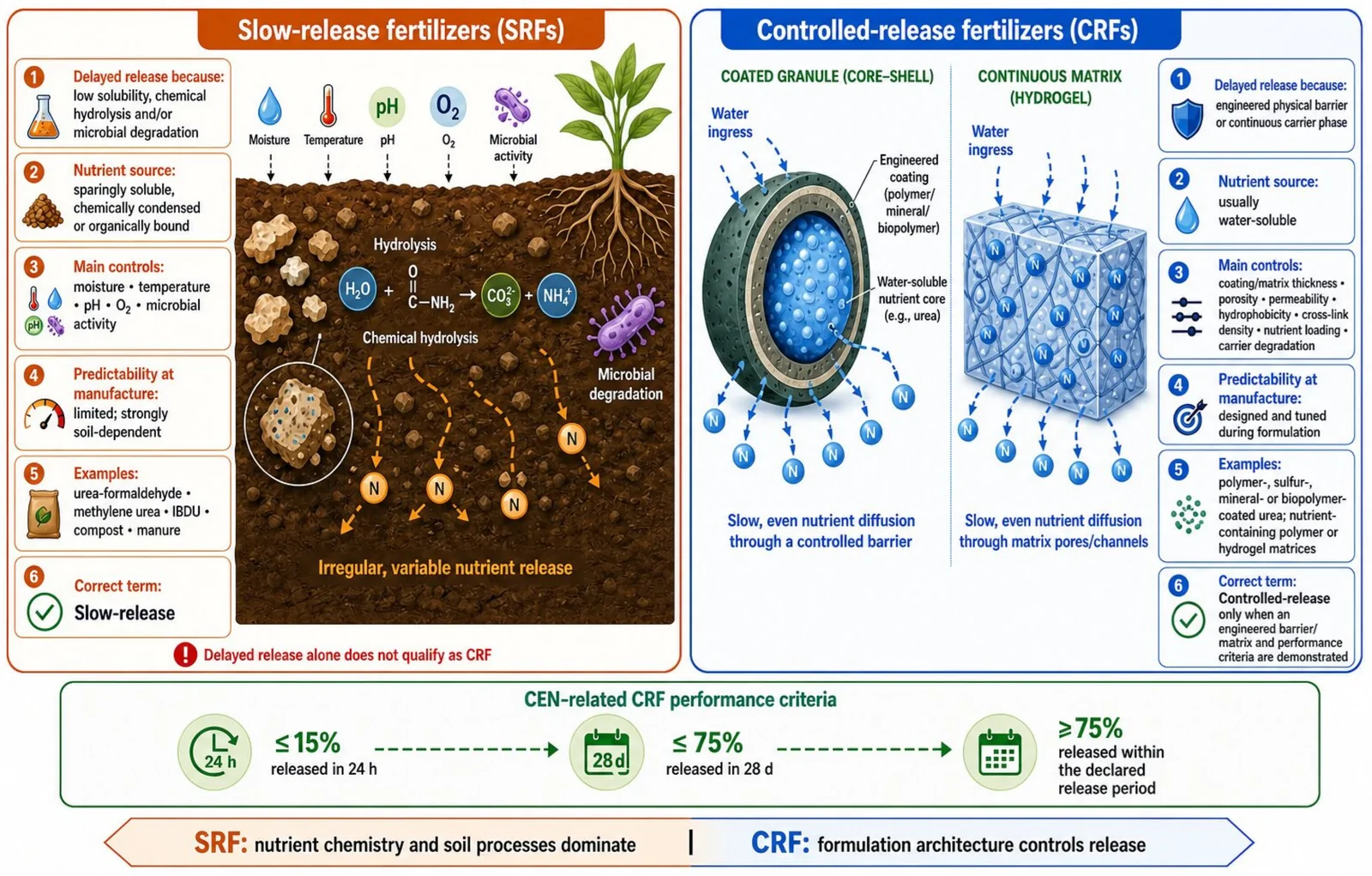
Schematic diagram: slow-release versus controlled-release fertilizers.

Slow-release fertilizers are products in which nutrient availability is delayed primarily because the nutrient source itself is sparingly soluble or must undergo chemical hydrolysis and/or microbial degradation before plant-available forms are released. Typical examples include urea-formaldehyde, methylene urea, isobutylidene diurea, crotonylidene diurea composts, manures, and other organic nutrient sources. Consequently, their nutrient-release rate is substantially dependent on soil temperature, moisture, pH, oxygen availability, and microbial activity. Such products can provide a prolonged nutrient supply, but their release profile cannot be precisely programmed at the time of manufacture [11]. Controlled-release fertilizers, in contrast, generally contain readily soluble nutrient sources whose release is delayed by an intentionally engineered physical structure, such as a coating, membrane, encapsulating shell, or continuous matrix. Their release profile is controlled by formulation variables including barrier thickness, porosity, hydrophobicity, permeability, cross-linking density, nutrient loading, and matrix degradation rate. Although soil conditions still influence CRF performance, the primary release-regulating function is incorporated into the product design rather than being determined predominantly by nutrient-source solubility or microbial transformation [8]. The distinction is also relevant from a performance perspective. The CEN criteria frequently used in the CRF literature define a controlled-release product by a release profile in which no more than 15% of nutrients are released within 24 h, no more than 75% within 28 d, and at least 75% within the declared release period. These thresholds should be treated as performance criteria assessed under specified testing conditions. They do not define the raw material, the manufacturing technology, or whether the product is coated or matrix-based. Therefore, a biodegradable formulation should not be labelled as a CRF solely because it releases nutrients more slowly than uncoated fertilizer; it should also demonstrate a designed release-controlling mechanism and satisfy the relevant release-performance criteria [12]. The criteria for distinguishing between SRFs and CRFs are presented in Table 1 and in Figure 1.

### 2.2. Coating-Based Systems and Matrix-Based Systems

From a classification standpoint, controlled-release fertilizers are most commonly divided into two main groups: coating-based systems and matrix-based systems [12]. These thresholds emphasize that CRF fertilizers are not merely “slow-release” fertilizers, but formulations designed to achieve a measurable and agronomically significant release profile [13]. This classification is technologically significant because the release mechanism, stability, environmental impact, and production cost of the fertilizer are closely linked to whether the nutrients are enclosed in an outer coating or dispersed within a carrier matrix [14,15]. Coated CRFs represent a classic family of core–shell systems in which the soluble fertilizer core (most commonly in the form of urea, NPK granules, monoammonium phosphate, diammonium phosphate, or similar mineral formulations) is surrounded by one or more outer layers that act as semipermeable barriers. Upon contact with soil moisture, water penetrates the coating, condenses on the core’s surface, and leads to its gradual dissolution; the resulting nutrient solutions then diffuse outward through pores, microvoids, and zones of swollen polymer [16,17]. Depending on the integrity and rheological properties of the coating, the release profile typically includes a distinct initial delay phase, followed by a quasi-constant release phase at a similar rate, and a final decay phase in which the diffusion rate decreases as the nutrient reserves in the core are depleted [18]. Based on coatings, CRF fertilizers can be divided into subclasses according to the chemical nature of the coating: sulfur-coated fertilizers, synthetic polymer-coated fertilizers, bio-based polymer-coated fertilizers, fertilizers with inorganic coatings, and fertilizers with multilayer and composite coatings [7]. Multilayer systems play a particularly important role in the latest research because they allow for the combination of complementary properties of different materials, for example, a highly hydrophobic inner barrier with an outer hydrogel layer or a mineral layer, which enables simultaneous control of nutrient diffusion, water penetration, mechanical stability, and, in some formulations, additional agronomic functions such as antifungal activity or targeted delivery of micronutrients [19].

Matrix-based controlled-release fertilizers constitute the second major class of CRFs. In these systems, nutrients are not enclosed solely within an outer coating; instead, fertilizer particles are embedded, dispersed, or immobilized within a continuous carrier phase that controls water permeation and nutrient transport. Release from matrix systems is primarily regulated by swelling, diffusion through the matrix, dissolution of dispersed nutrient regions, and, in some formulations, by gradual erosion or degradation of the carrier material [20]. Matrix materials can be hydrophobic or hydrophilic and include thermoplastic polymers, rubber-like materials, biodegradable polyesters, clays, hydrogels, and mixed organic-inorganic composites [19]. Hydrogels constitute a particularly significant subgroup within this class, as their three-dimensional networks absorb water, swell, and then regulate the release of nutrients while improving soil moisture retention [12]. Matrix systems can be divided into polymer-matrix fertilizers, hydrogel-based fertilizers, mineral-matrix fertilizers, and composite-matrix fertilizers, which combine polymeric and inorganic phases. Compared to coating-based systems, matrix fertilizers often require a higher proportion of carrier material to ensure effective release control, but they can offer simpler, solvent-free production methods, such as melt blending or extrusion, and can also incorporate additional functions, such as water retention or structural stabilization [21].

### 2.3. Other CRF Classifications

Material origin represents a separate classification axis and must not be confused with formulation architecture. Both coated and matrix CRFs can be prepared from synthetic polymers, sulfur-based materials, inorganic minerals, bio-based polymers, or organic–inorganic composites. Bio-based does not automatically mean biodegradable under field conditions, and biodegradability alone does not ensure compliance with CRF release criteria [22]. From a manufacturing technology perspective, CRFs can be grouped into coated granules, encapsulated particles, extruded composites, fertilizers embedded in a hydrogel matrix, and nanocomposite or superhydrophobic formulations. Although these categories partially overlap with the coating/matrix distinction, they are useful because they reflect the actual process pathway leading to a specific nutrient release mechanism and profile. Coated pellets are typically produced in tumbling or fluidized-bed processes involving the application of successive layers of barrier material; encapsulated particles are enclosed in polymeric or mineral microcapsules; while hydrogel and nanocomposite systems utilize three-dimensional polymer networks or nanofillers, respectively. Extruded composites are produced using extrusion, which is a promising method for manufacturing CRFs.

Finally, some authors propose a classification of CRFs based on nutrient release kinetics, distinguishing between formulations with essentially linear, parabolic, or sigmoidal release curves, and also taking into account the presence of successive phases: the delay phase, the steady-release phase, and the decay phase. Such kinetic classifications are of significant importance because the agronomic value of a controlled-release fertilizer depends not only on the retention time of nutrients in the system but, above all, on the degree to which the release profile aligns with the plants’ temporal nutrient requirements during the crop’s ontogenesis [3].

## 3. CRF Materials, Technologies and Nutrient-Release Mechanisms

### 3.1. Development of CRF Materials and Technologies

Although the CRF fertilizer market is still dominated by fertilizers coated with resins and thermoplastic polymers, current research is increasingly focusing on bio-based coatings, matrix systems, hydrogels, nanocomposites, and new processing technologies aimed at improving nutrient use efficiency while taking into account cost, release precision, and environmental stability. The first widely used materials in CRF fertilizers were sulfur and synthetic polymers. Urea coated with sulfur represented an important early technological step; however, brittleness, delamination, cracking, and inconsistent coating integrity often resulted in irregular nutrient release and a spike effect. These limitations accelerated the transition to synthetic polymer coatings, particularly polyurethane, polyethylene, polyolefin thermoplastics, alkyd resins, polyvinyl chloride, polysulfone, epoxy resin-based systems, and related materials, which allow for better control over release time, coating thickness, and membrane permeability [8,10].

Currently, the most advanced CRF technologies remain coating-based systems, in which soluble fertilizers, most commonly urea, ammonium nitrate, monoammonium phosphate, and diammonium phosphate, serve as the core. From a technological standpoint, the industry continues to rely heavily on rotary drum coating and fluidized bed coating, while laboratory-scale innovations increasingly involve chemical cross-linking, in situ polymerization, solvent casting, UV curing, multilayer encapsulation, and extrusion.

Fluidized-bed and rotary-drum coating are established routes for coated CRF production, but their industrial performance depends on stable rheological and drying conditions. In bio-based systems, fluctuations in viscosity may lead to non-uniform spraying, droplet coalescence, incomplete coverage, agglomeration, variable drying rates, or film defects. Uneven coating thickness directly affects water penetration and nutrient diffusion, resulting in different release periods among particles from the same batch. These methods are relatively simple and flexible but can be sensitive to insufficient humidity control and to the balance between spray rate, particle circulation, drying temperature, and bed moisture. Under inadequate conditions, porous or defective layers may form. Fluidized-bed coating can improve particle circulation and reduce coating-solution losses, but it requires narrow operating windows for inlet-air temperature, fluidization velocity, spray rate, nozzle design, and suspension viscosity. Highly viscous, particle-containing, or rapidly curing bio-based formulations can clog nozzles, produce irregular droplets, or create sticky particle surfaces, leading to agglomeration and coating defects [8].

Extrusion is currently a very promising method for CRF production, especially for matrix-based formulations in which the nutrient is dispersed in a continuous solid phase rather than protected solely by an outer coating. This method reduces solvent consumption, simplifies the processing, and enables the use of biodegradable polymers and mineral fillers to produce fertilizers with a tailored nutrient release profile [23]. The nutrient release profile in extruded CRFs depends on the internal architecture of the material, including the hydrophobicity of the matrix, filler dispersion, porosity, polymer crystallinity, and interfacial interactions between the nutrient and the carrier phase. From a technological standpoint, extrusion offers several significant advantages over traditional solvent-based coating methods. First, it eliminates or significantly reduces the use of organic solvents, which reduces emissions to the environment and can simplify subsequent drying steps. Second, extrusion combines mixing, shaping, and compaction into a single continuous process, making this method attractive for scaling up production and potentially reducing labor requirements and equipment complexity compared to multi-step coating processes. Third, extrusion facilitates the incorporation of a variety of fillers and waste-derived materials, including clays, starches, cellulose derivatives, biochar, and biodegradable polyesters, which aligns with current efforts to produce CRFs from renewable and circular economy-derived raw materials. Extrusion should therefore be considered one of the most promising unconventional methods for producing CRFs, especially for matrix-based, biodegradable, and hybrid organic–inorganic formulations [24]. Advantages, limitations, and scale-up requirements of CRF manufacturing methods are presented in Table 2.

**Figure 2:**
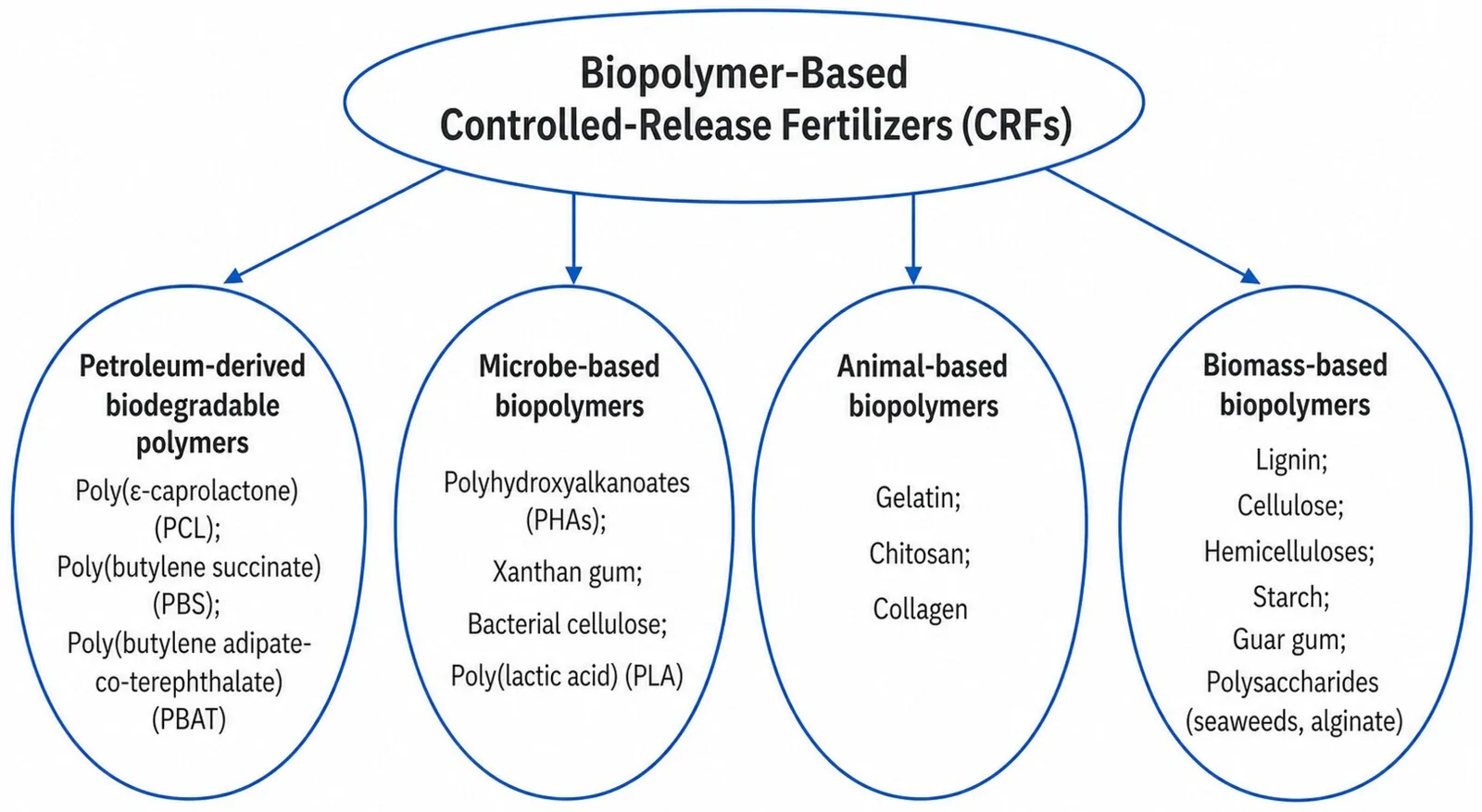
Examples of biopolymer-based CRFs.

Non-biodegradable coatings, such as polyolefins, polyvinylidene chloride, and related petroleum-derived polymers, can persist in the soil after nutrients have been released and contribute to the secondary accumulation of microplastics. This concern has become one of the strongest drivers of current research, steering material development toward biopolymers and other renewable raw materials capable of maintaining agronomic properties while improving degradability [25]. One of the major current trends is therefore the development of bio-based coating materials (Figure 2).

Among the most intensively studied substances are starch, cellulose, chitosan, lignin, alginate, carrageenan, natural rubber derivatives, vegetable oil-based polyurethanes, bio-oils, and combinations of these materials with mineral fillers or hydrophobic modifiers. These materials are attractive because they are renewable, often biodegradable, and can be chemically tailored through grafting, cross-linking, esterification, etherification, or surface modification to control water resistance, elasticity, swelling behavior, and nutrient diffusion [26].

Within the group of biopolymers, starch-based systems have attracted considerable attention owing to their wide availability, cost-effectiveness, and ease of chemical modification. Recent studies describe starch not only as a direct coating material but also as a component for hydrogel formation and as a component of composite films designed to improve mechanical stability and regulate nutrient release [27]. Cellulose and its derivatives, including carboxymethylcellulose, are equally important because they provide film-forming ability, biodegradability, and the potential to form cross-linked, water-resistant composite coatings [28]. Chitosan combines the ability to form water-retaining coatings with biocompatibility and ease of chemical modification [29]. Lignin has become one of the most promising bio-based CRF materials. It is no longer regarded merely as a low-value byproduct, but as a functional macromolecule whose hydrophobicity, aromatic structure, and reactive groups make it suitable for use in coated fertilizers, nanohybrids, bilayer systems, and polyurethane-like networks. Lignin-based systems have been combined with clay, alginate, paraffin, carbon black, polysiloxane, and epoxy resins to improve coating density, limit pore formation, reduce the rapid release of nutrients, and, in some cases, also reduce greenhouse gas emissions from the soil [30]. Table 3 presents a detailed breakdown of the biopolymers used in the production of CRFs [4].

Another important area of current research is matrix-based nutrient carriers, particularly hydrogels and composite matrices. Hydrogels based on polyvinyl alcohol, starch, soy protein, chitosan, alginate, carrageenan, cellulose derivatives, and mixed synthetic-natural systems are particularly attractive in drought-prone or degraded soils because they absorb water, swell, and then gradually release nutrients while maintaining local moisture [24]. Many of the latest controlled-release carriers are designed as integrated systems in which one layer provides mechanical strength, another introduces hydrophobicity, and a third is responsible for reactivity, nutrient enrichment, micronutrient delivery, or biological functionality. Examples include bilayer systems of biopolyurethane and alginate, lignin-based inner coatings reinforced with outer layers of paraffin or epoxy resin, castor oil-based polyurethane coatings modified with halloysite nanotubes or polysiloxanes, as well as cellulose–lignin or methylcellulose–lignin biocomposites intended for use in phosphate fertilizers [31,32]. In this field, there is also a shift away from formulations containing single nutrients toward multifunctional fertilizers. Some current CRF systems contain micronutrients such as zinc, copper, selenium, iron, and manganese, while others aim to combine controlled nutrient release with antifungal activity, water retention, reduced greenhouse gas emissions, or increased nutrient recovery from waste streams. This reflects a broader shift in development strategy: CRFs are increasingly being designed as multifunctional platforms for nutrient delivery, rather than as simple slow-release nitrogen carriers [33].

The use of CRFs is increasingly linked to broader concepts of process intensification and the circular economy. Substances currently being studied as coating precursors or matrix components include used cooking oil, apple tree branches, black locust sawdust, bagasse, cassava starch, lignocellulose from walnut wood, and lignin derived from industrial byproducts. At the same time, wastewater recovery technologies such as electrodialysis, struvite precipitation, biochar adsorption, and continuous-flow systems are being integrated with fertilizer production, suggesting that future CRFs may increasingly be derived from recovered nutrient streams rather than solely from primary raw materials [8]. In this context, compost may be an attractive option because it is derived from biomass residues and organic waste and provides benefits beyond the supply of nutrients, such as improved soil structure, increased microbial activity, better water retention, pH regulation, and long-term soil fertility [34]. From a technological standpoint, compost can be incorporated into CRF production in several ways. It can serve as the organic phase that supplies nutrients in hybrid fertilizers, which combine soluble inorganic nutrients with organic matter, thereby combining the immediate nutrient effect of mineral fertilizers with the benefits of improved soil properties resulting from organic additives. In such systems, compost itself does not provide controlled nutrient release; instead, it becomes part of the fertilizer’s core or composite structure, while a specially designed coating or matrix provides the actual control over nutrient release. Compost can also make an indirect contribution through the carbon-based materials it provides, particularly biochar, which has become an important carrier, adsorbent, and matrix in CRF systems [35]. Compost can also be incorporated into matrix-type CRFs, in which nutrients are dispersed in the solid phase rather than being protected solely by an outer coating. Matrix systems typically require relatively large amounts of excipients to effectively slow the release of nutrients; therefore, inexpensive, biodegradable solids derived from organic residues are of particular importance in this field. Due to its content of humified organic matter, fibrous structure, and reactive functional groups, compost could serve as a filler or blend component alongside biopolymers, clays, hydrogels, or waxes to modify porosity, water absorption capacity, and stability during degradation. However, such an application would require precise optimization, as excessive heterogeneity could compromise the reproducibility of nutrient release [36].

### 3.2. Advanced Technological Directions

Nanoscale fillers, including nanoclays, nanosilica, halloysite nanotubes, carbon-based nanomaterials, nanobiochar, layered double hydroxides, and polymeric nanoparticles, can improve CRF performance through several mechanisms. They can reduce coating porosity, increase tortuosity of diffusion pathways, reinforce polymer films, improve nutrient adsorption, and introduce responsive functions such as pH-dependent release or ion exchange. Nanostructured fillers can therefore improve nutrient loading, reduce early nutrient release, and enhance the mechanical stability of coatings or matrices. However, the laboratory advantages of nanoscale fillers are not automatically transferable to field or industrial conditions. Nanoparticles may agglomerate during storage, blending, coating, or extrusion, thereby reducing their effective surface area and creating defects rather than improving the barrier. Their performance also depends on uniform dispersion, particle-size distribution, surface modification, compatibility with the polymer phase, and stability under wetting–drying cycles. In soil, nanoparticles may interact with organic matter, clay minerals, microorganisms, roots, and co-contaminants in ways that are not represented by water-incubation tests. The industrialization threshold for nanocomposite CRFs should include reproducible large-scale production of the filler; low-cost and safe raw materials; stable dispersion without excessive surfactant or solvent use; compatibility with commercial coating or extrusion equipment; acceptable worker-safety and environmental-risk profiles; and multi-season field validation of nutrient-release performance and soil effects. Without these criteria, nanocomposite CRFs should be considered promising laboratory or pilot technologies rather than market-ready products [37].

In addition to nanotechnology, several less advanced but strategically important technological directions have also been identified. These include fertilizers based on metal–organic frameworks, plasma-assisted nitrogen fixation, continuous-flow liquid plasma systems, microfluidic sensors, and flow chemistry-assisted fertilizer production and recovery [38].

Superhydrophobic CRFs are designed to delay water ingress by combining low-surface-energy chemistry with micro- or nanoscale surface roughness. Such systems can be prepared using bio-based polyurethane, modified vegetable oils, polysiloxanes, paraffin waxes, halloysite nanotubes, nanosilica, carbon black, or other hydrophobic fillers. Their principal benefit is the reduction in early nutrient release with relatively low coating content. The principal field and industrial limitations are coating abrasion during transport and spreading, deterioration of surface roughness, loss of hydrophobicity during repeated wetting–drying cycles, incomplete curing, use of non-biodegradable additives, high nanofiller cost, and potential incompatibility with large-scale coating equipment. Consequently, superhydrophobic CRFs should be assessed by contact angle together with coating-thickness distribution, abrasion resistance, compression strength, resistance to wetting–drying and temperature cycling, release behaviour in multiple soils, biodegradation profile, and cost per hectare [7].

Metal–organic frameworks are porous coordination materials constructed from metal nodes and organic linkers. Their high surface area, tunable pore structure, and capacity to host multiple nutrients make them attractive as controlled-release nutrient carriers. MOFs can potentially regulate nutrient release through pore confinement, ligand exchange, ion exchange, framework dissolution, and pH-dependent degradation. However, MOF fertilizers remain an early-stage technology. Their raw-material costs depend on the metal precursor, ligand type, solvent, synthesis temperature, purification requirements, and nutrient-loading procedure. Some frameworks require expensive organic ligands, high-purity metal salts, organic solvents, and energy-intensive synthesis. In addition, framework stability may be altered by soil pH, moisture, carbonate concentration, competing ions, and microbial activity. The potential release of metals, ligands, or synthesis residues must be assessed before agricultural application. Fe-based MOFs are among the most promising candidates because iron is a plant nutrient and some Fe-MOF systems have been synthesized at both laboratory and pilot scales. However, comparable product yields at these scales do not yet demonstrate field-scale economic viability. The industrialization threshold for MOF fertilizers should include low-cost or solvent-free synthesis, defined metal and ligand purity, reproducible nutrient loading, safe degradation products, stability during storage and field application, contaminant monitoring, and multi-site field trials comparing MOF fertilizers with established mineral, sulfur-coated, polymer-coated, and bio-based CRFs [39].

Nutrient recovery from wastewater can reduce dependence on primary nutrient resources and link CRF technology with circular-economy systems. Relevant recovery routes include electrodialytic recovery of ammonium and phosphate, reverse osmosis, struvite precipitation, adsorption on biochar or activated carbon, and recovery in fluidized-bed reactors. Recovered products can be used directly as fertilizers, nutrient concentrates, mineral components, or ingredients in CRF matrices and coatings. The technical limitation is that recovered nutrient streams may be heterogeneous. Depending on the source, they can contain salts, heavy metals, pathogens, residual organic matter, pharmaceuticals, industrial chemicals, and other micropollutants. Therefore, the key industrial challenge is to convert variable waste streams into a standardized product with predictable nutrient content, low contaminant concentrations, consistent physical properties, and regulatory compliance. For example, electrodialytic recovery can concentrate ammonium and phosphate, but membrane fouling, electricity consumption, pH adjustment, and brine management affect cost. Fluidized-bed struvite recovery can achieve high phosphate recovery, but requires stable control of pH, magnesium supply, circulation rate, and hydraulic retention time. The commercial threshold is reached only when nutrient-recovery efficiency, contaminant control, energy use, chemical consumption, transport logistics, and fertilizer-market value are favourable simultaneously [40].

Although these approaches are not yet standard methods for producing CRFs, they indicate that this field is moving beyond the mere design of materials toward integrated systems that combine nutrient synthesis, smart delivery, sensors, and recovery technologies. The integration of CRFs with precision agriculture should be understood as adaptive nutrient management rather than as direct real-time control of a fertilizer particle after it has been applied. A conventional CRF has a predetermined release profile that cannot be instantly modified by a sensor. Sensors can nevertheless improve CRF performance by supporting product selection, site-specific application, release verification, and corrective nutrient management. This requires reliable sensor calibration in complex soils, data transmission, interoperability between devices and farm-management systems, robust crop nutrient-demand models, and transparent decision algorithms. It also requires economic validation because sensor deployment is beneficial only when the value of reduced nutrient losses, labour savings, or yield improvement exceeds the cost of sensors, communications, data processing, and technical support [6].

### 3.3. Nutrient-Release Mechanisms

Nutrient release from controlled-release fertilizers should not be interpreted as a single universal process. The release profile is determined by the formulation architecture, the physical and chemical properties of the release-controlling material, the nutrient source, and environmental conditions. In practice, nutrient release depends to a large extent on temperature, humidity, pH, microbial activity, nutrient solubility, coating thickness, pore structure, and the physical integrity of the coating or matrix. Therefore, kinetic models should be selected according to the dominant release mechanism rather than applied indiscriminately to all CRF systems [41]. The effect of environmental factors on the three phases of nutrient release is shown in Table 4 [42,43].

**Figure 3:**
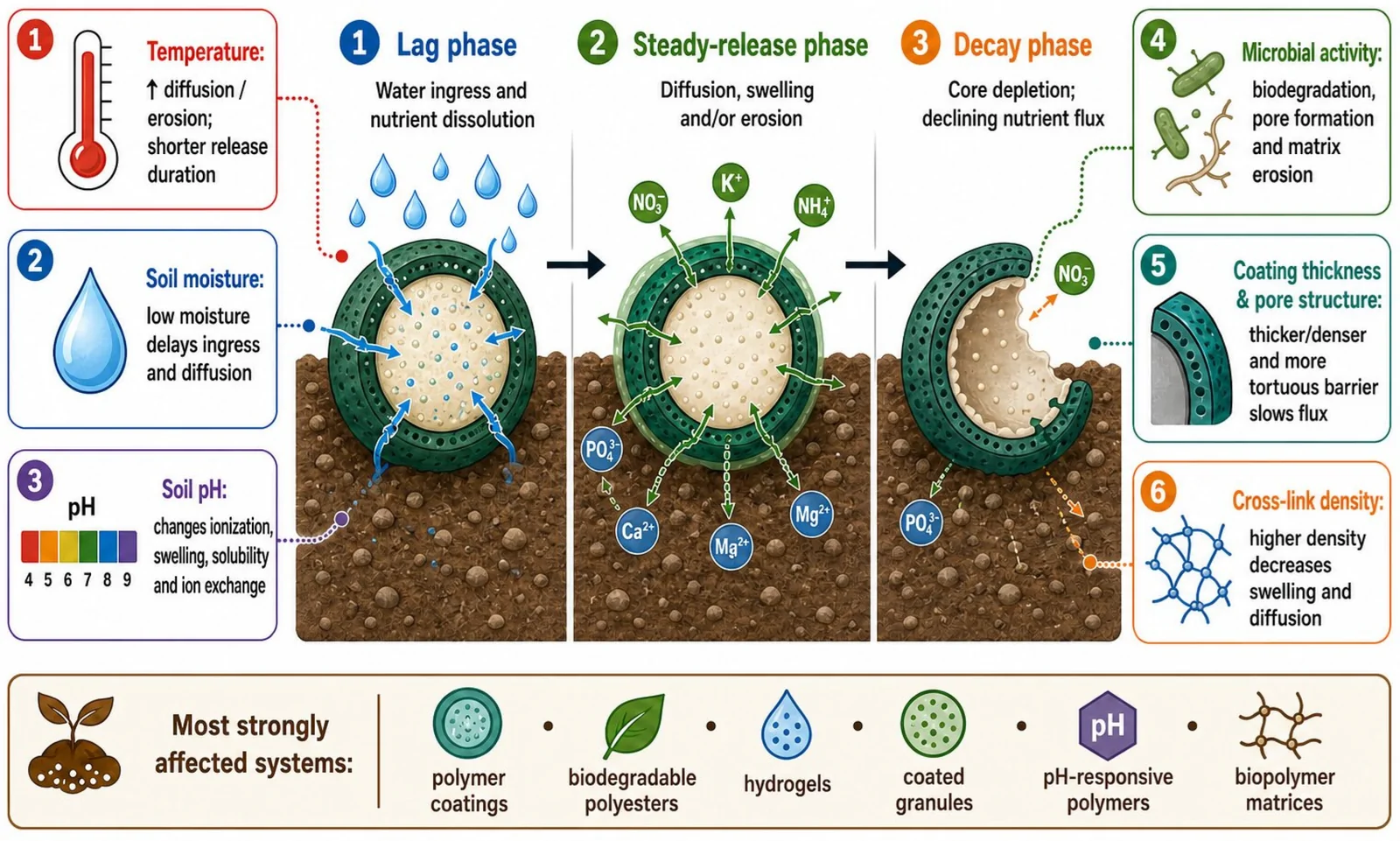
Environmental factors regulate nutrient-release phases in CRFs.

For many coated CRFs, nutrient release can be described by three consecutive phases: a lag phase, a steady-release phase, and a decay phase. During the lag phase, soil water penetrates the coating or matrix, dissolves the soluble nutrient source, and generates an internal nutrient solution. In coated granules, this process increases osmotic pressure within the core. During the steady-release phase, the nutrient solution diffuses outward through micropores, microvoids, or swollen polymer domains, generally under a concentration-gradient driving force. In the decay phase, the nutrient concentration within the granule decreases because the core is progressively depleted; consequently, the concentration gradient and nutrient flux decline (Figure 3).

**Figure 4:**
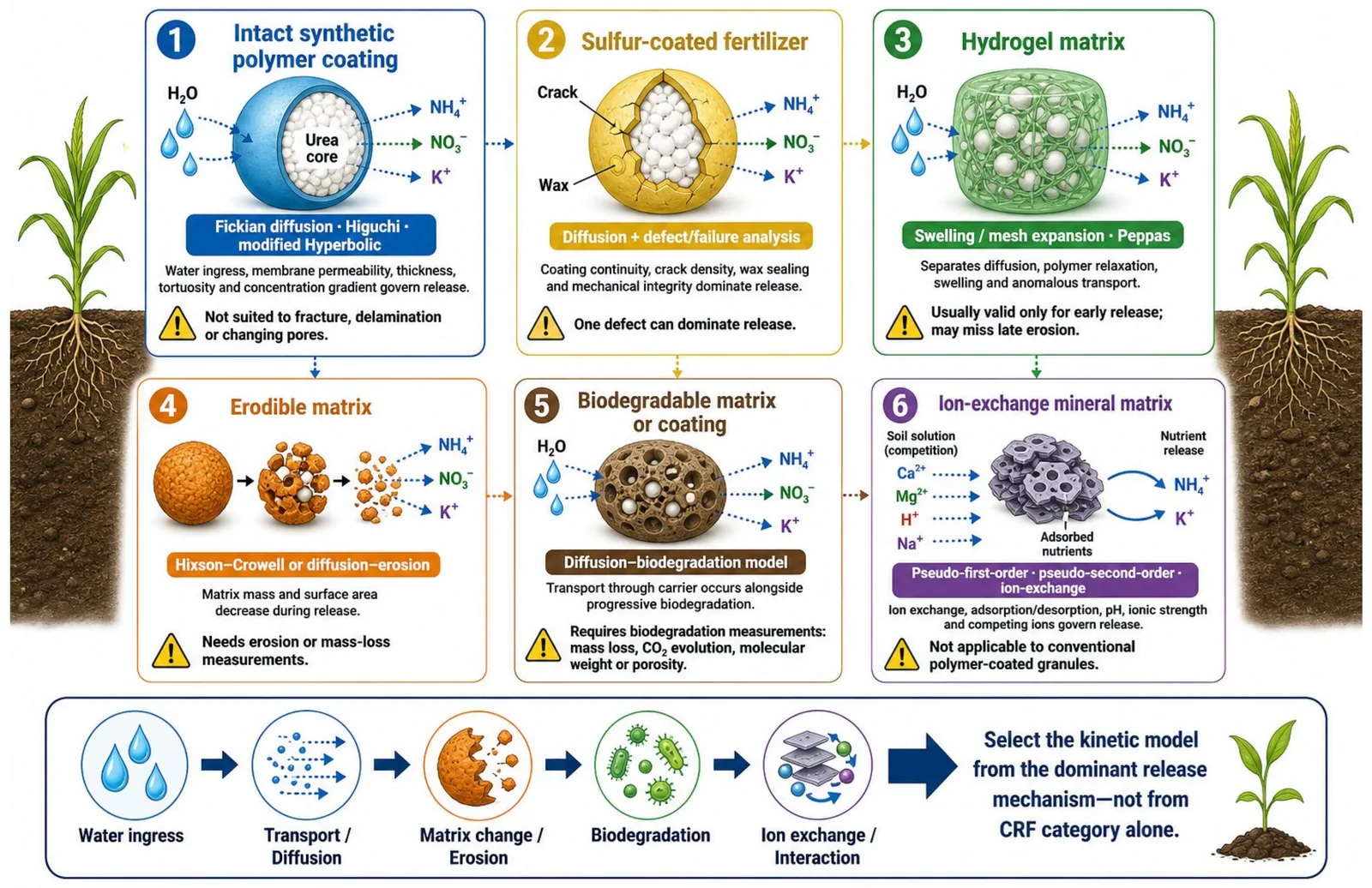
Mechanism-based models for nutrient release from CRFs.

The duration and shape of these three phases differ substantially among sulfur-coated fertilizers, synthetic-polymer-coated fertilizers, hydrogels, and biodegradable matrices. Therefore, the use of the terms “lag phase,” “steady-release phase,” and “decay phase” should not imply that all CRFs exhibit identical kinetics or that each phase is regulated by the same process. Mechanism-based models for nutrient release from CRFs are presented in Figure 4 and in Table 5.

## 4. Agricultural Applications of CRFs

CRFs are increasingly used in a wide range of agricultural systems because they improve the timing between nutrient release and crop demand. Their agricultural applications range from large-scale cereal and oilseed crops to horticultural crops, orchards, nurseries, seedling systems, drought-prone soils, and even highly specialized controlled-growth systems.

The agronomic performance of controlled-release fertilizers cannot be compared reliably without considering experimental scale, cropping environment, fertilizer rate, application method, trial duration, soil properties, and the type of conventional fertilizer used as a comparator. Results from pot experiments, soil-column tests, and field trials should therefore not be pooled as if they represented identical agronomic conditions. Pot and greenhouse trials generally provide well-controlled evidence on nutrient release, plant uptake, and short-term nutrient loss, whereas field trials capture the combined effects of climate, soil heterogeneity, irrigation or rainfall, crop development, and fertilizer-management practices (Table 6).

One of the most important areas of CRF application is field crop production, particularly cereals, rice-based crops, corn, canola, wheat, and sugarcane. In such systems, CRFs are mainly used to replace or supplement conventional base fertilization and multiple split applications, which reduces labor requirements and improves nutrient use efficiency through a single application or a limited number of applications [4]. Improved nutrient-use efficiency (NUE) has been shown to be one of the most clearly observed benefits of using CRFs. By regulating the availability of nutrients over time, CRFs reduce the mismatch between the rate at which fertilizer dissolves and the rate at which plants take it up, thereby increasing the percentage of applied nutrients that are absorbed by the crop. In practice, this means that CRFs are often used not only to increase the fertilizer application rate but also to improve fertilization efficiency, especially where nitrogen losses have agronomic and environmental significance [54]. In a 65-day pot study, biochar-coated urea increased nitrogen-use efficiency by about 20% compared with uncoated urea in oilseed rape, while also reducing nitrogen losses and improving soil nitrogen status. Likewise, controlled-release formulations have been reported to improve nutrient uptake and recovery in maize when compared with uncoated combined NPK fertilizers. These observations support the broad conclusion that CRFs are particularly useful where fertilizer efficiency is constrained by rapid nutrient mobility or weak retention in soil [55].

Another important area of application in agriculture is horticulture, which includes the cultivation of vegetables, fruits, and high-value specialty crops. CRFs are particularly attractive in these systems due to the high value of the crops, intensive nutrient management, and the economic importance of quality characteristics. Controlled-release fertilization increased tomato yields and nitrogen use efficiency compared to conventional fertilization [56]. Other studies have shown improvements in soluble sugar content, vitamin C, lycopene, starch accumulation, seed protein content, chlorophyll content, and fruit quality in crops such as tomatoes, corn, apples, and peaches. These results suggest that the agronomic value of controlled-release fertilizers lies not only in maintaining biomass production but also in improving the nutritional value and market quality of harvested products [8]. The use of CRFs in corn cultivation has also been described [46]. The reported results include increased grain yields, greater starch accumulation, higher vitamin C levels in some studies, and significant improvements when using controlled-release urea coated with sulfur and urease. Maize grain yield increased by 21.9–65.5%, while grain starch content increased by 18.1–76.6% when CRFs were applied compared to conventional practices [46]. Although these benefits vary depending on the formulation and growing conditions, they indicate that corn is one of the crops in which CRF fertilizers have demonstrated particularly high agronomic potential.

Rice is another important crop for the application of controlled-release fertilizers in agriculture, especially since flooded and seasonally flooded cropping systems are prone to nitrogen losses and low fertilizer utilization rates. Controlled-release urea can increase rice yields, reduce ammonia volatilization, and improve nitrogen utilization in floodplain rice cropping systems, provided it is applied properly. This is important because rice cultivation often combines high fertilizer requirements with a high risk of nitrogen loss to the environment, making controlled nutrient delivery particularly attractive [45]. Among the field applications described are also oilseed and rapeseed crops. Field experiments with early-maturing rapeseed showed that CRF increased seed yield and net profit compared to soluble fertilizer, while improving nutrient uptake and overall fertilizer use efficiency [55].

Fruit tree cultivation represents another important application niche. The literature describes the beneficial effects of using CRFs in perennial fruit tree crops, such as apple trees [50] and peach trees [52], where nutrient requirements vary seasonally, and fruit quality has a major impact on profitability. In the case of apple trees, the use of CRFs was associated with an increase in trunk diameter, plant height, new shoot growth, and chlorophyll content [50], whereas controlled-release fertilizers used in peach cultivation delayed leaf senescence and improved the nutritional value of the fruit during storage, as well as fruit yield and quality [52].

CRFs are also increasingly being studied in vegetable crops and protected cultivation systems, as they can provide a more balanced supply of nutrients in intensive production environments. Vegetables respond positively to controlled nutrient delivery by improving plant growth, nutrient uptake, and reducing the risk of fertilizer-induced damage resulting from localized salt accumulation or excessive nutrient concentrations during the early growth stage [11]. CRFs are also valuable in nursery and seedling production. It has been reported that placing a CRF carrier within the root ball or near the root zone promotes early seedling growth, and the more gradual nutrient release profile from CRF carriers reduces the risk of osmotic stress associated with highly soluble fertilizers. As a result, CRFs are useful in transplanting systems, ornamental plant nursery production, and other situations where plant establishment and early vigor are important [57].

Another agricultural application involves dry, degraded, or coarse-textured soils, where the ability to retain nutrients is inherently low. Systems based on CRF matrices and containing hydrogels are particularly useful under such conditions, as they can combine nutrient release with water retention, local moisture conservation, and improvement of soil physical properties. The reported environmental benefits resulting from the use of CRFs are just as significant as the agronomic benefits. The use of CRFs is associated with reduced nutrient leaching, a lower risk of surface runoff, reduced ammonia volatilization, better nitrogen retention in the soil, and a lower likelihood of eutrophication compared to conventional soluble fertilizers. These benefits are particularly important in regions where excessive fertilizer use, groundwater contamination, and agricultural greenhouse gas emissions are major challenges to sustainable development [58]. It has been shown that zeolite-coated urea reduced nitrogen leaching by 65% [41], while urea coated with thermoplastic resin, sulfur, and polyolefin reduced ammonia volatilization by 82.7%, 78.4%, and 69.4%, respectively. It has also been noted that the use of CRFs can reduce nitrogen losses by 30–40% compared to traditional fertilizers, thereby improving both soil fertility and environmental protection [59].

In addition to reducing nutrient loss, CRFs can improve soil quality and conditions in the root zone. The gradual release of nutrients from CRFs can stimulate microbial activity, indirectly improve soil structure through a better nutrient balance and humus formation, and reduce the sharp fluctuations in nutrient concentrations that often accompany soluble fertilizers. Some matrix-based systems containing biochar also contribute directly to increased water-holding capacity, improved soil properties, and nutrient adsorption at the soil-fertilizer interface [4,8]. Agonomic safety is also reported as a benefit. Because CRF fertilizers help avoid the very high local concentrations of ions that can occur immediately after the application of conventional fertilizers, they can reduce osmotic stress and fertilizer burn, particularly in seedlings and sensitive horticultural crops. This is one of the reasons why sigmoidal or otherwise moderated release profiles are often considered preferable, as they better match the rate at which plants take up nutrients and reduce the likelihood of phytotoxicity during the early growth stage.

Controlled-release fertilizers can provide operational and economic benefits in agriculture. Because nutrients are supplied over a longer period, it may be necessary to apply fertilizers less frequently, which can reduce labor requirements and simplify nutrient management compared to multiple, split applications of soluble fertilizers [8]. Another area of application, though still relatively limited, is the controlled cultivation of plants in closed or atypical environments. The use of Florikan controlled-release fertilizers has been documented in NASA’s “Veggie” plant cultivation system on the International Space Station, where a steady supply of nutrients was ensured through the use of CRF fertilizers embedded in a fired ceramic rooting medium. Although this is not a conventional agricultural environment, it illustrates the versatility of controlled-release fertilizer technology wherever a long-term supply of nutrients is required with minimal maintenance [8].

## 5. Major Scientific, Technical, Environmental, and Economic Issues

Despite significant progress in the design of CRFs, their wider implementation continues to face a number of interrelated scientific, technical, environmental, and economic challenges. The main scientific challenge in developing CRFs is the difficulty of synchronizing nutrient release with crop demand under realistic and variable soil conditions [8]. In principle, CRFs are designed to deliver nutrients at a set rate and according to a specific schedule; however, in practice, nutrient release depends on many factors. As a result, a release profile that appears optimal under laboratory conditions may not remain optimal under field conditions, where weather, irrigation systems, soil type, and root zone activity are constantly changing. This discrepancy is particularly significant because plants’ nutrient requirements are inherently dynamic and crop-specific. Nutrient uptake typically follows a sigmoid curve, characterized by low demand during the rooting phase, a sharp increase in demand during the vegetative and reproductive growth periods, and a decline in demand as maturity approaches. CRFs are expected to mimic this profile; however, even when using the same coating material, changes in coating concentration, thickness, and exposure to environmental factors can significantly affect the delay time, the steady-state release period, and the decay profile. Therefore, one of the ongoing scientific challenges is to predict and adjust the release of nutrients in a way that remains physiologically appropriate for various crops and production systems [23].

One of the remaining unresolved scientific questions is the long-term impact of materials used in CRF on soil fauna and flora, as well as on soil processes. It has been shown that nutrient release and the degradation of certain materials are regulated by microbial activity; however, microbial abundance and function can vary significantly depending on soil aeration, waterlogging, drought, and temperature. As a result, a formulation that performs well in one environment may release nutrients too slowly or too quickly in another, potentially causing nutrient deficiencies, nutrient loss, or reduced agronomic yield [3]. Therefore, one of the ongoing scientific challenges is to predict and adjust the release of nutrients in a way that remains physiologically appropriate for various crops and production systems [52].

Sulfur-based coatings are a good example of these technical challenges. Sulfur is attractive because it is inexpensive, biodegradable, and can also serve as a secondary nutrient; however, its brittleness, tendency to crack, poor adhesion, delamination, and sensitivity to phase changes can lead to uncontrolled nutrient leakage or rapid release. To overcome these limitations, sulfur-coated fertilizers often require the addition of waxes, conditioning agents, sealants, or other modifiers, which complicates production and may increase costs [14]. Petroleum-based polymer coatings provide more precise control over drug release but come with other technical limitations. Commonly used thermoplastic and thermosetting coatings can provide a long release time; however, their preparation may require the use of chlorinated solvents, complex coating procedures, and careful control of humidity, temperature, cross-linking, and coating thickness. Even relatively minor defects, such as pores, cracks, or surface imperfections causing adhesion, which arise during polycondensation, can disrupt the continuity of the layer and significantly alter the release profile of nutrients [6].

Production reproducibility poses another significant technical challenge. Industrial production requires uniform coating thickness, a constant polymer-to-fertilizer ratio, and homogeneous coverage of a large number of granules; however, this is difficult to achieve on a large scale. Problems such as uneven coating, porous layers, fluctuations in reactor moisture content, and defects arising in pan or drum coating systems can cause significant variability between individual granules, which reduces product reliability. Scaling up the production of biodegradable and bio-based CRF materials remains a particular challenge. Although bio-based materials are increasingly promoted as sustainable alternatives, many of these systems still face limitations related to insufficient mechanical strength, excessive hydrophilicity, too rapid a release of nutrients, limited shelf life, manual or periodic coating procedures, or high production costs for biopolymer raw materials. Consequently, the technical challenge lies not only in replacing fossil-based polymers but also in developing bio-based systems that match them in terms of nutrient release precision and performance under agricultural conditions [47].

The environmental issues associated with CRF fertilizers are paradoxical, as the very products intended to reduce nutrient pollution may themselves impose new burdens on the environment. The most frequently highlighted environmental concern is the persistence of synthetic polymer residues in the soil [60]. After nutrients are released, petroleum-based coating materials may remain in the soil and accumulate over time; estimates indicate that in some cases, the amount of polymer residues can reach as high as 50 kg/ha/year. The accumulation of these residues is discussed as a source of so-called “white pollution” and as a pathway leading to secondary microplastic contamination in agricultural soils. This issue is significant not only because of the visible accumulation of residues but also because the long-term ecological consequences remain insufficiently understood. Coating residues can impair soil permeability and generate degradation products of uncertain ecotoxicological significance, while some synthetic polymer production processes involve the use of volatile organic compounds or toxic solvents. Environmental concerns also extend to coating ingredients and additives. Some alkyd or resin-based systems require drying agents that may contain toxic metals, while some coating application processes rely on chlorinated hydrocarbons or other solvents that are undesirable from the perspectives of health and emissions [61]. This means that the environmental assessment of CRFs should cover not only nutrient retention in the field, but also the impact of raw materials, additives, solvents, energy consumption, and the fate of residues throughout the entire life cycle. Bio-based alternatives alleviate some of these concerns, but do not eliminate them entirely. Many biopolymer coatings are more environmentally attractive because they are renewable and potentially biodegradable; however, some degrade too quickly to maintain a synchronized release of nutrients throughout the growing cycle. Consequently, the environmental challenge is twofold: the coatings should not persist as pollutants, but they should also not lose their structural integrity so quickly that nutrient losses reoccur [60].

A complete environmental assessment of CRF coatings should integrate agronomic function with material fate and ecological effects (Table 7). First, raw materials, monomers, catalysts, cross-linkers, plasticizers, solvents, nanofillers, and energy requirements should be evaluated during production. Second, the fertilizer should be tested for nutrient-release conformity, including burst release, tailing, nutrient losses, and crop response under relevant soil conditions. Third, after nutrient depletion, the residual coating should be monitored for mass loss, molecular-weight change, fragmentation, microplastic generation, biodegradation, mineralization, and transport within the soil profile [62,63].

Ecotoxicological assessment should include soil microbial biomass, bacterial and fungal community composition, soil enzyme activities, respiration, nitrogen-cycling processes, earthworm survival and reproduction, and where relevant, other soil fauna. The assessment should also consider whether coating residues alter the sorption, desorption, or transport of metals, pesticides, and hydrophobic organic pollutants. Finally, a life-cycle assessment should compare the environmental benefits resulting from reduced nutrient losses with potential burdens related to material production, energy consumption, solvent emissions, residual accumulation, microplastic formation, and end-of-life fate.

Economic barriers remain one of the most significant constraints to the widespread use of CRFs. The production of CRFs is typically more expensive than that of conventional fertilizers, as it requires additional coating materials, more complex processing, stricter quality control, and, in some cases, costly polymers, additives, or solvents. Even if agronomic performance is higher, the higher purchase price may limit their use, especially in low-margin cropping systems or in regions where fertilization decisions are heavily influenced by price. The economic viability of using CRFs depends on whether the additional costs are offset by increased yields, improved fertilizer efficiency, labor savings, improved product quality, or reduced environmental impacts. Positive economic outcomes are possible because CRFs can reduce the need for repeated fertilizer applications in multiple doses and can increase net profit. However, the same literature also indicates that when the cost of CRFs rises to about twice that of conventional fertilizers, the net economic benefit may disappear or even become negative [64]. The scale of production and the complexity of the process also affect the economic profile of CRFs. Some promising formulations are tested using dip coating, manual wrapping, specialized cross-linking steps, or laboratory spray-coating methods, which may be difficult or costly to implement in continuous industrial production. Where scaling up production is difficult, manufacturing costs remain high, and consistency between batches may still be insufficient to inspire market confidence. The economic issue therefore concerns not only the nominal price of the fertilizer but also uncertainty regarding its consistent performance under real-world conditions. The data presented thus suggest that the future of CRF carriers depends less on a single breakthrough discovery and more on integrated progress in the fields of materials science, nutrient release modeling, life-cycle assessment, agronomic validation, and cost reduction [4].

## 6. Conclusions and Proposed Directions for Development

### 6.1. Conclusions

Controlled-release fertilizers represent an important strategy for improving the synchronization between nutrient availability and crop demand, thereby reducing nutrient losses, improving nutrient-use efficiency, and potentially lowering the environmental burden associated with conventional soluble fertilizers. However, CRF performance is not determined by coating or matrix material alone. It depends on the interaction among product architecture, nutrient source, release mechanism, coating thickness, porosity, hydrophobicity, degradation behaviour, soil conditions, crop demand, application strategy, manufacturing reproducibility, and environmental fate.

The current evidence shows that CRFs can improve yield, crop quality, nutrient recovery, and nitrogen-use efficiency under appropriate conditions. At the same time, their benefits cannot be generalized across crops, soils, climates, and production systems. Sulfur-based coatings may be cost-effective but remain vulnerable to cracking and heterogeneous release. Synthetic polymer coatings can provide predictable release but may persist in soil and contribute to microplastic accumulation. Bio-based materials can reduce dependence on fossil-derived polymers, but their rapid swelling, biodegradation, mechanical weakness, or variable composition may lead to premature nutrient release. Therefore, sustainable CRF development should not be defined merely as the replacement of synthetic polymers with bio-based materials; it should aim to achieve synchronized nutrient release, safe end-of-life behaviour, field reliability, scalable manufacture, and economic feasibility simultaneously.

### 6.2. Short-Term Feasible Priorities

The most immediate research priority is to improve the reliability of currently available CRF architectures. This includes optimizing coating thickness, cross-linking density, hydrophobicity, porosity, filler content, matrix composition, and mechanical strength to reduce burst release, premature cracking, and prolonged tailing. In particular, bio-based formulations should be designed to maintain structural integrity during the intended nutrient-release period and subsequently degrade without forming persistent or harmful residues.

A second short-term priority is to improve the reproducibility and scalability of manufacturing. Future studies should report material specifications, feedstock origin, coating mass, coating-thickness distribution, particle-size distribution, viscosity, moisture content, curing conditions, matrix homogeneity, production yield, rejection rate, and nutrient-release variation among production batches. Process development should focus on solvent-reduced or solvent-free coating, extrusion, pelletization, UV curing, and other scalable methods that can maintain product quality while reducing energy use and manufacturing cost.

A third priority is to establish a more rigorous agronomic evidence base. CRF performance should be tested against appropriate conventional fertilizer controls at equivalent nutrient rates and under defined soil and crop conditions. Research should report not only yield and nutrient uptake, but also nitrogen-use efficiency, ammonia volatilization, nitrate leaching, nitrous oxide emissions, nutrient-release kinetics, residual coating mass, soil microbial response, and economic return. Pot and water-incubation studies remain useful for mechanism identification, but they should be complemented by field trials that account for variable weather, soil heterogeneity, irrigation regime, and crop phenology.

### 6.3. Long-Term Frontier Directions

Long-term CRF research should move toward multifunctional nutrient-delivery systems capable of combining controlled release with water retention, nutrient recovery, micronutrient supply, reduced greenhouse-gas emissions, pathogen suppression, and improved soil health. Promising directions include multilayer bio-based coatings, biodegradable polymer–mineral composites, biochar-containing matrices, superhydrophobic surfaces, nanostructured barriers, and stimuli-responsive materials that respond to temperature, moisture, pH, or root-zone conditions.

Advanced systems should be developed cautiously. Nanoscale fillers, nanoclays, nanosilica, halloysite nanotubes, metal–organic frameworks, microfluidic encapsulation, plasma-derived nutrients, and wastewater-recovered fertilizer components may improve nutrient loading and release precision. However, their development should include assessment of raw-material cost, manufacturing complexity, field stability, ecotoxicity, residual-material fate, and industrial feasibility from the beginning. High laboratory performance should not be considered sufficient evidence of practical value without multi-season field validation and life-cycle assessment.

A further long-term direction is the integration of CRFs with precision agriculture. Conventional CRFs have predetermined release profiles and cannot be instantaneously reprogrammed after application. However, sensors, remote sensing, weather forecasts, crop-growth models, and decision-support systems can improve CRF selection, variable-rate placement, in-season diagnosis of nutrient mismatch, and corrective fertilization. The future role of CRFs should therefore be viewed as part of adaptive nutrient-management systems rather than as an isolated fertilizer technology.

### 6.4. Fundamental Methodological Gaps

The first major research gap is the absence of standardized long-term field-trial protocols for CRFs. Many studies use different nutrient-release media, temperatures, soil types, fertilizer rates, crop species, study durations, and performance indicators, making direct comparison difficult. A harmonized protocol should include at least one full crop cycle and preferably multi-season or multi-year evaluation, multiple soil textures and climatic conditions, conventional-fertilizer controls, and standardized measurements of yield, nutrient uptake, NUE, nutrient loss, greenhouse-gas emissions, residual-coating mass, and soil biological responses.

The second gap is the lack of unified biodegradation-testing standards specifically designed for CRF coatings and matrices. A material should not be described as biodegradable solely on the basis of mass loss in water, laboratory compost, or short soil burial tests. Biodegradation assessment should include mass loss, molecular-weight reduction, mineralization, carbon dioxide evolution, residue characterization, microplastic or fragment formation, soil microbial response, and the relationship between degradation rate and nutrient-release profile. Testing should be conducted in representative soils with controlled and reported moisture, temperature, pH, oxygen status, and microbial activity.

Another research gap is the absence of dedicated lifecycle assessment systems for CRFs. Conventional LCA approaches should be adapted to include CRF-specific categories: nutrient-use efficiency, nitrate leaching, ammonia volatilization, nitrous oxide emissions, residual polymer accumulation, microplastic generation, biodegradation products, solvent use, curing energy, nanomaterial inputs, transport, fertilizer application frequency, and end-of-life fate. The resulting assessment should compare the environmental benefit gained through reduced nutrient loss with burdens related to material production and residual environmental exposure.

## Figures and Tables

**Table 1:** Criteria for distinguishing between SRFs and CRFs.

Criterion	Slow-Release Fertilizers (SRFs)	Controlled-Release Fertilizers (CRFs)
Primary reason for delayed release	Low nutrient solubility; chemical hydrolysis and/or microbial degradation	Engineered physical barrier or continuous carrier phase
Nutrient source	Usually sparingly soluble, chemically condensed, or organically bound	Usually water-soluble nutrient source
Main controlling factors	Soil moisture, temperature, pH, oxygen availability, and microbial activity	Coating or matrix thickness, porosity, permeability, hydrophobicity, cross-link density, nutrient loading, and carrier degradation
Predictability at manufacture	Limited; strongly soil-dependent	Can be designed and tuned during formulation
Typical examples	Urea-formaldehyde, methylene urea, IBDU, compost, manure	Polymer-, sulfur-, mineral-, or biopolymer-coated urea; nutrient-containing polymer or hydrogel matrices
CEN-related CRF performance criterion	Not sufficient to classify a product as CRF	≤15% released in 24 h; ≤75% released in 28 d; ≥75% within the declared release period
Correct terminology	“Slow-release”	“Controlled-release” only when an engineered barrier/matrix and performance criteria are demonstrated

**Table 2:** Advantages, limitations, and scale-up requirements of CRF manufacturing methods.

Manufacturing Method	CRF Architecture	Advantages at Laboratory Scale	Main Laboratory Limitations	Industrial Advantages	Principal Industrial Barriers	Priority Quality-Control Parameters
Rotary-drum or pan coating	Coated granule/core–shell	Simple equipment; flexible formulation changes; suitable for screening different coatings	Uneven manual spraying; limited control of thickness; solvent evaporation; batch variability	Mature and relatively simple technology; suitable for large granules and multilayer application	Uneven coating thickness; humidity sensitivity; agglomeration; prolonged drying; granule breakage; variable coating efficiency	Granule size distribution, coating mass, thickness distribution, bed moisture, spray rate, drying temperature, abrasion resistance
Fluidized-bed coating	Coated granule/core–shell	Efficient particle circulation; improved heat and mass transfer; reduced solution losses; thin-film application	Narrow operating window; nozzle clogging; difficult scale-down of airflow and droplet behaviour	Continuous or semi-continuous operation; good coating uniformity when properly controlled	Viscosity variation, poor atomization, sticky surfaces, nozzle fouling, particle agglomeration, high energy demand for air handling and drying	Feed viscosity, solids content, droplet size, spray rate, inlet/outlet air temperature, air velocity, agglomerate fraction, coating continuity
Spouted-bed coating	Coated granule/core–shell	Good contact between particles and spray; useful for some particle-size ranges	Bed instability; limited operating data for bio-based high-viscosity coatings	Potentially lower coating-solution losses and efficient circulation	Stable spouting is difficult with variable particle size or sticky formulations; scale-up correlations may be uncertain	Particle circulation, pressure drop, spray distribution, coating mass, agglomeration, residence-time distribution
Dip coating/immersion	Coated granule/core–shell	Low-cost proof-of-concept; simple for small batches; useful for material screening	Excess coating, poor thickness control, long drying time, solvent use	Limited suitability for high-throughput manufacture	Difficult continuous operation; high solvent demand; inconsistent coating-to-core ratio; poor reproducibility	Solution viscosity, withdrawal speed, coating mass, drying profile, residual solvent
Solvent casting	Film, coating, hydrogel, or composite layer	Precise preparation of films and multilayer structures; useful for mechanistic studies	Slow drying; solvent residue; limited sample size; poor representation of industrial conditions	Mainly useful for specialized products rather than commodity fertilizer	High solvent-recovery demand, emissions, low throughput, thickness variation, difficult automation	Solvent content, drying rate, film thickness, porosity, residual solvent, adhesion
In situ polymerization/chemical cross-linking	Coating, hydrogel, or matrix	Strong adhesion; tunable cross-link density; can produce responsive materials	Reaction-sensitive; initiator and catalyst dependence; difficult reaction control	Potential for durable coatings and functional hydrogels	Control of reaction heat, monomer safety, curing uniformity, batch reproducibility, catalyst residues	Conversion degree, gel fraction, cross-link density, residual monomer, viscosity profile, mechanical strength
UV curing/roller coating	Thin bio-based coating	Rapid curing; low solvent use; potentially continuous	Requires optimized photoinitiator and light exposure; limited penetration through opaque systems	High-speed continuous coating and reduced drying burden	Uniform irradiation, shadowing, surface oxygen inhibition, viscosity control, lamp maintenance, incomplete cure	UV dose, curing time, coating thickness, conversion degree, tackiness, adhesion, release profile
Melt extrusion and pelletization	Matrix CRF/extruded composite	Solvent-free; continuous; simultaneous mixing and shaping; suitable for biodegradable polymers and fillers	Thermal degradation; limited residence-time control; small batches may not predict industrial mixing	High throughput; integrates blending, compaction, shaping, and pellet production	Poor nutrient/filler dispersion, feeder variability, torque fluctuations, die fouling, thermal degradation, pellet-density variation	Feed moisture, feeder accuracy, temperature profile, torque, die pressure, residence time, pellet density, nutrient homogeneity
Compression molding/heat pressing	Matrix tablet, granule, or coating	Simple production of test specimens; controlled pressure and thickness	Slow, discontinuous, low throughput; poor simulation of continuous production	Suitable for specialized tablets or small-volume products	Manual handling, limited throughput, heat-transfer gradients, variability between press cycles	Temperature, pressure, dwell time, thickness, density, mechanical strength, release kinetics
Multilayer coating/sequential encapsulation	Multilayer core–shell CRF	Combines different functions; enables tailored release and multifunctionality	Many steps; interlayer compatibility difficult to assess	Can deliver sophisticated controlled-release profiles	Delamination, adhesion failure, different curing rates, layer-thickness variability, increased cost and process time	Interfacial adhesion, layer thickness, coating continuity, swelling mismatch, wetting–drying stability, abrasion resistance

**Table 3:** Modification requirements and nutrient-control limitations of biopolymer-based CRFs.

Biopolymer Group	Main Limitation for Nutrient Control	Typical Modification Strategy	Remaining Drawback to State in Manuscript
Starch and thermoplastic starch	High water sensitivity, rapid biodegradation, weak mechanical integrity, and possible early burst release	Grafting, esterification, cross-linking, blending with hydrophobic polymers, siloxane treatment, mineral nanofillers	Stronger hydrophobization may reduce biodegradation; release may remain variable under wet field conditions
Cellulose and cellulose derivatives	Hydrophilicity and swelling can increase water penetration; plant cellulose may require purification and shows source-dependent properties	Etherification, esterification, grafting, cross-linking, nanocellulose reinforcement, blending with lignin or biochar	Hydrogels may release nutrients too rapidly to meet CRF criteria; bacterial cellulose production remains costly
Chitosan and chitin derivatives	Rapid biodegradation, pH-sensitive swelling, limited water resistance, and possible burst release in hydrogel systems	Ionic or covalent cross-linking, grafting, clay/nanoclay incorporation, wax or hydrophobic outer layer	Strong cross-linking can reduce biodegradability and may make films brittle; release changes with soil pH and temperature
Lignin and lignocellulosic materials	Feedstock heterogeneity, variable functional-group composition, film defects, and inconsistent barrier performance	Liquefaction, graft copolymerization, polyurethane networks, blending with paraffin, epoxy, siloxane, carbon black, or clay	Hydrophobicity can delay nutrient release excessively; some modifiers may compromise the environmental advantage of a bio-based formulation
Alginate, carrageenan, agar, and seaweed polysaccharides	High swelling and ion-sensitive gel structure; limited long-term barrier capacity	Multivalent-ion cross-linking, grafting, blending with carbonaceous materials, multilayer systems	Swelling can cause burst release and make release strongly dependent on pH, ionic strength, and moisture
Protein-based biopolymers	Moisture sensitivity, relatively weak films, denaturation during processing, and batch variability	Cross-linking, plasticizers, blending with polysaccharides or hydrophobic phases	Greater cross-link density may reduce flexibility and produce heterogeneous nutrient diffusion pathways
Microbial polyesters: PHA/PHB/PHBV	High cost, processing-window constraints, brittleness of highly crystalline grades, and biodegradation-dependent release	Copolymerization, plasticization, blending, mineral fillers, melt extrusion	Faster microbial degradation can cause premature nutrient loss; slower degradation may not match crop nutrient demand
PLA, PBS, PCL and related biodegradable polyesters	Often require blending or additives to tune hydrophobicity and degradation; some are not fully bio-derived	Melt blending, plasticization, mineral fillers, reactive compatibilization, solvent-free coating or extrusion	Degradation rate may be too slow or strongly condition-dependent; higher cost and thermal processing may limit scale-up
Biochar-based composites	Variable feedstock chemistry, pore structure, adsorption capacity, and nutrient desorption behaviour	Surface activation, nutrient impregnation, blending with polymers, clay, or hydrogels	Excessive nutrient adsorption can reduce immediate availability; reproducibility depends on pyrolysis conditions and feedstock

**Table 4:** Influence of environmental factors on the phases of nutrient release.

Environmental Factor	Lag Phase	Steady-Release Phase	Decay Phase	Systems Most Strongly Affected
Temperature	Higher temperature increases water mobility and polymer-chain mobility, generally shortening the lag phase	Increases diffusion coefficient and may accelerate matrix erosion or hydrolysis	Causes earlier nutrient-core depletion and a shorter total release duration	Polymer coatings, biodegradable polyesters, hydrogels, SRFs
Soil moisture	Low moisture delays water ingress and prolongs the lag phase	Limited water availability reduces diffusion and swelling	May extend tailing because the nutrient core is incompletely dissolved	All CRFs; especially hydrogels and coated granules
Soil pH	Can alter ionization and swelling of pH-responsive polymers	Changes hydrogel mesh size, nutrient solubility, mineral dissolution, adsorption/desorption, and ion exchange	May modify hydrolysis and the residual nutrient-binding capacity of the matrix	Chitosan, alginate, carboxymethylcellulose, mineral matrices, pH-responsive systems
Microbial activity	Usually minor for intact non-biodegradable coatings; may be important for biodegradable barriers	Increases biodegradation, pore formation, and matrix erosion	Can reduce residual matrix integrity and accelerate late-stage release	Starch, cellulose, chitosan, lignin, PHA, PLA, PBS, PCL, urea-formaldehyde systems
Coating thickness and pore structure	Thicker, denser coatings extend lag time	Lower pore volume and higher tortuosity reduce nutrient flux	Delays the onset of depletion and extends the decay phase	Sulfur, synthetic polymer, multilayer coatings
Cross-link density	Can delay water uptake in hydrogels and coatings	Higher cross-link density generally decreases swelling and diffusion	May prolong residual release but can also create brittle structures	Hydrogels, polyurethane, cross-linked polysaccharide systems

**Table 5:** Models for nutrient release from CRFs.

CRF Architecture and Dominant Mechanism	Recommended Kinetic Approach	Key Interpretation	Main Limitation
Intact synthetic polymer coating	Fickian diffusion model; Higuchi model; modified hyperbolic model	Nutrient release is mainly determined by water ingress, membrane permeability, coating thickness, pore tortuosity, and concentration gradient	Does not adequately describe fracture, delamination, or changing pore structure
Sulfur-coated fertilizer	Diffusion model combined with defect/failure analysis	Release depends strongly on coating continuity, crack density, wax sealing, and mechanical integrity	A single defect may dominate release and invalidate ideal diffusion assumptions
Hydrogel matrix	Korsmeyer–Peppas/Ritger–Peppas model	Distinguishes diffusion, polymer relaxation, swelling, and anomalous transport	Often valid only for the initial proportion of release; may not capture late erosion
Erodible matrix	Hixson–Crowell model or diffusion–erosion combined model	Matrix mass and surface area progressively decrease during release	Requires erosion or mass-loss measurements in addition to nutrient-release data
Biodegradable matrix or coating	Combined diffusion–biodegradation model	Nutrient release depends on both transport through the carrier and progressive biodegradation	Requires independent measures of biodegradation, such as mass loss, CO_2_ evolution, molecular-weight decrease, or changes in porosity
Ion-exchange mineral matrix	Pseudo-first-order, pseudo-second-order, or ion-exchange models	Release depends on ion exchange, adsorption/desorption, pH, ionic strength, and competing ions	Not directly applicable to conventional polymer-coated granules

**Table 6:** The efficiency of CRFs under various experimental conditions.

Trial Type and Conditions	Crop	Fertilizer Treatment and Category	Duration Time	Results Compared to Conventional Fertilization	Ref.
Field cultivation	Maize	Controlled release urea	Growing season	5.3% yield improvement	[44]
Field cultivation; paddy flooded	Rice	Sulfur and urease coated controlled release fertilizer	Growing season	Yield increased by approximately 55–68%; Grain protein content rose from 5.8% to 14.9%;	[45]
Field cultivation	Maize	Controlled release urea	Growing season	Grain yield rose markedly, by about 21.9–65.5%; Starch content in the grain increased by roughly 18.1–76.6%; The concentration of vitamin C was enhanced;	[46]
Pot experiment	Amaranthus plant	PVA/soy-protein hydrogel with urea; bio-based matrix CRF	28 days	Leaf nitrogen content was 58% higher than in the control pots, indicating more efficient use of supplied nutrients;	[47]
Field cultivation; paddy flooded	Rice	Controlled-release urea	Growing season	0.1–12.4% yield improvement;	[48]
Pot experiment	Tomato	Canola-oil/sulfur polysulfide composite; sulfur-containing bio-based CRF	10 weeks	The plants exhibited deeper green foliage, greater height, and a higher number of fruits during the 10-week period;	[49]
Field cultivation; orchard conditions	Apple	Various types of CRFs	Growing season	Yield increased by 8.82%; Significantly improved the growth of apple trees in trunk diameter, plant height, new shoot growth and chlorophyll content;	[50]
Field cultivation	Cotton plant	Epoxy-resin-coated urea and polymer-coated KCl; synthetic polymer CRF	Growing season	PCU and PCPC fertilizers increased cotton yield more effectively than urea (35.3% for PCU and 40.7% for PCPC);	[51]
Field cultivation; orchard conditions	Peach trees	Bag-controlled release fertiliser	Growing season	Yield increased by 21.35%; Improved NUE by 37.73%; Improved shoot growth in young peach trees, increasing trunk growth by 26.22%;	[52]
Field cultivation; red/yellow soil; southern China	Rapeseed	Various types of CRFs	2 years trial	Increased seed yields of early ripening rapeseed by 14.51% compared to soluble fertilisers; N accumulation and usage efficiency rose by 13.66% and 9.74%, respectively.	[53]

**Table 7:** Environmental risks, degradation limitations, and mitigation strategies for CRF coating materials.

Material Group	Environmental Benefit	Main Degradation or Persistence Concern	Risk of Uncontrolled Nutrient Release	Main Factors Controlling Behaviour	Recommended Mitigation Strategy
Petroleum-based polymers: polyolefins, PE, PVC, PVDC, epoxy resins	High mechanical strength and precise release control	Persistent residual shells; fragmentation into microplastics and nanoplastics; potential long-term accumulation	Usually low initially, but cracks and defects can cause localized burst release	Coating thickness, defects, tillage, weathering, UV exposure, oxidation, soil abrasion	Reduce coating mass; use solvent-free manufacture; improve shell integrity; assess residual mass and microplastic formation
Sulfur coatings	Low cost; sulfur also acts as a secondary nutrient	Cracking, delamination, acidic effects after sulfur oxidation	High if cracks or incomplete coating are present	Coating thickness, sulfur phase transitions, wax sealants, soil moisture, microbial oxidation	Use modified sulfur, sealants, waxes, and quality control for coating continuity
Starch and thermoplastic starch	Renewable, low cost, biodegradable	High water sensitivity, rapid swelling, rapid microbial degradation, low mechanical strength	High in wet soils because coating damage can cause burst release	Soil moisture, temperature, microbial activity, cross-linking, blending ratio	Cross-linking, grafting, hydrophobic modification, mineral fillers, multilayer coating
Chitosan	Biodegradable, pH-responsive, film-forming, biocompatible	pH-dependent swelling; relatively rapid biodegradation; variable strength	Moderate to high if swelling or biodegradation is rapid	Soil pH, temperature, cross-link density, clay or wax additives	Ionic/covalent cross-linking; nanoclay or mineral reinforcement; hydrophobic outer layer
Lignin and lignocellulosic materials	Renewable industrial by-product; hydrophobicity; good film formation	Feedstock heterogeneity; variable degradation; potential slow persistence if highly hydrophobic	Moderate; may be too slow or too fast depending on structure and modification	Biomass source, extraction process, functional groups, blend composition, soil microbial activity	Standardize lignin feedstock; combine with cellulose, clay, alginate, or biochar; avoid non-degradable modifiers where possible
Alginate, carrageenan, and other seaweed polysaccharides	Renewable, non-toxic, good water retention	Swelling and ion-sensitive network destabilization	High where water uptake or ionic exchange causes rapid swelling	Soil moisture, pH, ionic strength, cross-linking ions	Multivalent-ion cross-linking, carbonaceous fillers, multilayer structures
PHA, PHB, PHBV, PLA, PBS, PCL	Potentially biodegradable; tunable mechanical properties	Degradation rate may be too slow or too rapid depending on crystallinity and soil conditions	Moderate if microbial degradation or hydrolysis occurs before crop nutrient demand is met	Temperature, moisture, oxygen, microbial activity, crystallinity, molecular weight	Polymer blending, plasticization, mineral fillers, field-specific biodegradation testing
Hybrid bio-based coatings	Can combine strength, hydrophobicity, and biodegradability	Environmental profile may be compromised by synthetic cross-linkers, waxes, epoxy resins, nanofillers, or persistent additives	Depends on compatibility and degradation sequence of the components	Interfacial stability, component ratio, layer order, additive fate	Perform component-specific fate and toxicity assessment; prioritize biodegradable, low-toxicity modifiers

## Data Availability

No new data were created or analyzed in this study. Data sharing is not applicable to this article.

## References

[1] Knorr D., Khoo C.S.H., Augustin M.A. (2018). Food for an Urban Planet: Challenges and Research Opportunities. Front. Nutr..

[2] van Dijk M., Morley T., Rau M.L., Saghai Y. (2021). A Meta-Analysis of Projected Global Food Demand and Population at Risk of Hunger for the Period 2010–2050. Nat. Food.

[3] Lawrencia D., Wong S.K., Low D.Y.S., Goh B.H., Goh J.K., Ruktanonchai U.R., Soottitantawat A., Lee L.H., Tang S.Y. (2021). Controlled Release Fertilizers: A Review on Coating Materials and Mechanism of Release. Plants.

[4] Ravindran A., Manivannan A.C., Kandaiah R., Kulanthaisamy M., Indirathankam S.C., Nachimuthu G., Panneerselvan L., Palanisami T. (2025). Advancements and Challenges in Controlled-Release Fertilisers: An Approach to Integrate Biopolymer-Based Strategies. Ind. Crops Prod..

[5] Yu K., Fang X., Zhang Y., Miao Y., Liu S., Zou J. (2021). Low Greenhouse Gases Emissions Associated with High Nitrogen Use Efficiency under Optimized Fertilization Regimes in Double-Rice Cropping Systems. Appl. Soil Ecol..

[6] Asadu C.O., Ezema C.A., Ekwueme B.N., Onu C.E., Onoh I.M., Adejoh T., Ezeorba T.P.C., Ogbonna C.C., Otuh P.I., Okoye J.O. (2024). Enhanced Efficiency Fertilizers: Overview of Production Methods, Materials Used, Nutrients Release Mechanisms, Benefits and Considerations. Environ. Pollut. Manag..

[7] Singh A.K. (2025). Bio-Based Materials for Fabrication of Controlled-Release Coated Fertilizers to Enhance Soil Fertility: A Review. Next Mater..

[8] Govil S., Van Duc Long N., Escribà-Gelonch M., Hessel V. (2024). Controlled-Release Fertiliser: Recent Developments and Perspectives. Ind. Crops Prod..

[9] Chien S.H., Prochnow L.I., Cantarella H. (2009). Chapter 8 Recent Developments of Fertilizer Production and Use to Improve Nutrient Efficiency and Minimize Environmental Impacts. Advances in Agronomy.

[10] Yokamo S., Milinga A.S., Suefo B. (2023). Alternative Fertilization Approaches in Enhancing Crop Productivity and Nutrient Use Efficiency: A Review. Arch. Agric. Environ. Sci..

[11] Morgan K.T., Cushman K.E., Sato S. (2009). Release Mechanisms for Slow- and Controlled-Release Fertilizers and Strategies for Their Use in Vegetable Production. HortTechnology.

[12] Mansouri H., Ait Said H., Noukrati H., Oukarroum A., Ben Youcef H., Perreault F. (2023). Advances in Controlled Release Fertilizers: Cost-Effective Coating Techniques and Smart Stimuli-Responsive Hydrogels. Adv. Sustain. Syst..

[13] Gutiérrez C.A., Ledezma-Delgadillo A., Juárez-Luna G., Neri-Torres E.E., Ibanez J.G., Quevedo I.R. (2022). Production, Mechanisms, and Performance of Controlled-Release Fertilizers Encapsulated with Biodegradable-Based Coatings. ACS Agric. Sci. Technol..

[14] Blouin G.M., Rindt D.W., Moore O.E. (1971). Sulfur-Coated Fertilizers for Controlled Release: Pilot Plant Production. J. Agric. Food Chem..

[15] Liu Y.-H., Wang T.-J., Qin L., Jin Y. (2008). Urea Particle Coating for Controlled Release by Using DCPD Modified Sulfur. Powder Technol..

[16] Vroman I., Tighzert L. (2009). Biodegradable Polymers. Materials.

[17] Davidson D., Gu F.X. (2012). Materials for Sustained and Controlled Release of Nutrients and Molecules to Support Plant Growth. J. Agric. Food Chem..

[18] Ibrahim S., Nawwar G.A.M., Sultan M. (2016). Development of Bio-Based Polymeric Hydrogel: Green, Sustainable and Low Cost Plant Fertilizer Packaging Material. J. Environ. Chem. Eng..

[19] Witt T., Robinson N., Palma A.C., Cernusak L.A., Pratt S., Redding M., Batstone D.J., Schmidt S., Laycock B. (2024). Evaluating Novel Biodegradable Polymer Matrix Fertilizers for Nitrogen-Efficient Agriculture. J. Environ. Qual..

[20] Seddighi H., Shayesteh K., Omrani N., Es’haghi P. (2024). Fertilizers Coating Methods: A Mini Review of Various Techniques. Chem. Res. Technol..

[21] Valle S.F., Giroto A.S., Ribeiro C. (2023). Unveiling the Role of Polysulfide Matrices in Phosphate Diffusion for Controlled Release Fertilizers. ACS Appl. Polym. Mater..

[22] El-Aziz M.E.A., Salama D.M., Morsi S.M.M., Youssef A.M., El-Sakhawy M. (2022). Development of Polymer Composites and Encapsulation Technology for Slow-Release Fertilizers. Rev. Chem. Eng..

[23] Bi S., Barinelli V., Sobkowicz M.J. (2020). Degradable Controlled Release Fertilizer Composite Prepared via Extrusion: Fabrication, Characterization, and Release Mechanisms. Polymers.

[24] Song Y., Ma L., Duan Q., Xie H., Dong X., Zhang H., Yu L. (2024). Development of Slow-Release Fertilizers with Function of Water Retention Using Eco-Friendly Starch Hydrogels. Molecules.

[25] Kassem I., Ablouh E.-H., El Bouchtaoui F.-Z., Jaouahar M., El Achaby M. (2024). Polymer Coated Slow/ Controlled Release Granular Fertilizers: Fundamentals and Research Trends. Prog. Mater. Sci..

[26] Chojnacka K., Moustakas K., Witek-Krowiak A. (2020). Bio-Based Fertilizers: A Practical Approach towards Circular Economy. Bioresour. Technol..

[27] Wang C., Song S., Du L., Yang Z., Liu Y., He Z., Zhou C., Li P. (2023). Nutrient Controlled Release Performance of Bio-Based Coated Fertilizer Enhanced by Synergistic Effects of Liquefied Starch and Siloxane. Int. J. Biol. Macromol..

[28] Tian H., Zhang L., Sun X., Cui J., Dong J., Wu L., Wang Y., Wang L., Zhang M., Liu Z. (2022). Self-Healing Modified Liquefied Lignocellulosic Cross-Linked Bio-Based Polymer for Controlled-Release Urea. Ind. Crops Prod..

[29] Han Y., Chen S., Yang M., Zou H., Zhang Y. (2020). Inorganic Matter Modified Water-Based Copolymer Prepared by Chitosan-Starch-CMC-Na-PVAL as an Environment-Friendly Coating Material. Carbohydr. Polym..

[30] Abbas A., Wang Z., Zhang Y., Peng P., She D. (2022). Lignin-Based Controlled Release Fertilizers: A Review. Int. J. Biol. Macromol..

[31] Kusumastuti Y., Istiani A., Rochmadi, Purnomo C.W. (2019). Chitosan-Based Polyion Multilayer Coating on NPK Fertilizer as Controlled Released Fertilizer. Adv. Mater. Sci. Eng..

[32] Liu X., Wu L., Zhou W., Hu L., Lv J., Du W. (2022). Environment-Friendly Bio-Based Controlled-Release Phosphate Fertilizer with Waste Kitchen Oil as Coating Material: Preparation, Characterization, and Mechanisms. J. Environ. Manag..

[33] Ma X., Zhang S., Yang Y., Tong Z., Shen T., Yu Z., Xie J., Yao Y., Gao B., Li Y.C. (2023). Development of Multifunctional Copper Alginate and Bio-Polyurethane Bilayer Coated Fertilizer: Controlled-Release, Selenium Supply and Antifungal. Int. J. Biol. Macromol..

[34] Zhang T., Li H., Yan T., Shaheen S.M., Niu Y., Xie S., Zhang Y., Abdelrahman H., Ali E.F., Bolan N.S. (2023). Organic Matter Stabilization and Phosphorus Activation during Vegetable Waste Composting: Multivariate and Multiscale Investigation. Sci. Total Environ..

[35] Ayilara M.S., Olanrewaju O.S., Babalola O.O., Odeyemi O. (2020). Waste Management through Composting: Challenges and Potentials. Sustainability.

[36] Naher U.A., Biswas J.C., Maniruzzaman M., Khan F.H., Sarkar M.I.U., Jahan A., Hera M.H.R., Hossain M.B., Islam A., Islam M.R. (2021). Bio-Organic Fertilizer: A Green Technology to Reduce Synthetic N and P Fertilizer for Rice Production. Front. Plant Sci..

[37] Beig B., Niazi M.B.K., Sher F., Jahan Z., Malik U.S., Khan M.D., Américo-Pinheiro J.H.P., Vo D.-V.N. (2022). Nanotechnology-Based Controlled Release of Sustainable Fertilizers. A Review. Environ. Chem. Lett..

[38] Puač N., Gherardi M., Shiratani M. (2018). Plasma Agriculture: A Rapidly Emerging Field. Plasma Process. Polym..

[39] Terrón D., Sanromán A., Pazos M. (2025). Metal–Organic Frameworks: Next-Generation Materials for Environmental Remediation. Catalysts.

[40] Ye Y., Ngo H.H., Guo W., Chang S.W., Nguyen D.D., Zhang X., Zhang J., Liang S. (2020). Nutrient Recovery from Wastewater: From Technology to Economy. Bioresour. Technol. Rep..

[41] Mihok F., Macko J., Oriňak A., Oriňaková R., Kovaľ K., Sisáková K., Petruš O., Kostecká Z. (2020). Controlled Nitrogen Release Fertilizer Based on Zeolite Clinoptilolite: Study of Preparation Process and Release Properties Using Molecular Dynamics. Curr. Res. Green Sustain. Chem..

[42] Hou D., He J., Lü C., Sun Y., Zhang F., Otgonbayar K. (2013). Effects of Environmental Factors on Nutrients Release at Sediment-Water Interface and Assessment of Trophic Status for a Typical Shallow Lake, Northwest China. Sci. World J..

[43] Jariwala H., Santos R.M., Lauzon J.D., Dutta A., Wai Chiang Y. (2022). Controlled Release Fertilizers (CRFs) for Climate-Smart Agriculture Practices: A Comprehensive Review on Release Mechanism, Materials, Methods of Preparation, and Effect on Environmental Parameters. Environ. Sci. Pollut. Res..

[44] Zhang W., Liang Z., He X., Wang X., Shi X., Zou C., Chen X. (2019). The Effects of Controlled Release Urea on Maize Productivity and Reactive Nitrogen Losses: A Meta-Analysis. Environ. Pollut..

[45] Khan A.Z., Ali B., Afzal M., Wahab S., Khalil S.K., Amin N., Ping Q., Qiaojing T., Zhou W. (2015). Effects of Sulfur and Urease Coated Controlled Release Urea on Dry Matter Yield, n Uptake and Grain Quality of Rice. J. Anim. Plant Sci..

[46] Dong Y.J., He M.R., Wang Z.L., Chen W.F., Hou J., Qiu X.K., Zhang J.W. (2016). Effects of New Coated Release Fertilizer on the Growth of Maize. J. Soil Sci. Plant Nutr..

[47] KP A.A., Saeed P.A., Manholi S., Sujith A. (2024). Polyvinyl Alcohol-Soy Protein Isolate Hydrogels: Controlled Release of Fertilizer and Matrix Nutrients for Sustainable Agriculture. J. Clean. Prod..

[48] Xu Q., Dai L., Shang Z., Zhou Y., Li J., Dou Z., Yuan X., Gao H. (2023). Application of Controlled-Release Urea to Maintain Rice Yield and Mitigate Greenhouse Gas Emissions of Rice–Crayfish Coculture Field. Agric. Ecosyst. Environ..

[49] Mann M., Kruger J.E., Andari F., McErlean J., Gascooke J.R., Smith J.A., Worthington M.J.H., McKinley C.C.C., Campbell J.A., Lewis D.A. (2019). Sulfur Polymer Composites as Controlled-Release Fertilisers. Org. Biomol. Chem..

[50] Li J., Liu Y., Tang Y., Shao J., Xu T., Ma R., Jiang Y., Cheng D. (2022). Optimizing Fertilizer Management Based on Controlled-Release Fertilizer to Improve Yield, Quality, and Reduce Fertilizer Application on Apples. J. Soil. Sci. Plant Nutr..

[51] Yang X., Geng J., Li C., Zhang M., Tian X. (2016). Cumulative Release Characteristics of Controlled-Release Nitrogen and Potassium Fertilizers and Their Effects on Soil Fertility, and Cotton Growth. Sci. Rep..

[52] Zhang Y., Luo J., Peng F., Xiao Y., Du A. (2021). Application of Bag-Controlled Release Fertilizer Facilitated New Root Formation, Delayed Leaf, and Root Senescence in Peach Trees and Improved Nitrogen Utilization Efficiency. Front. Plant Sci..

[53] Tian C., Zhou X., Liu Q., Peng J., Wang W., Zhang Z., Yang Y., Song H., Guan C. (2016). Effects of a Controlled-Release Fertilizer on Yield, Nutrient Uptake, and Fertilizer Usage Efficiency in Early Ripening Rapeseed (*Brassica napus* L.). J. Zhejiang Univ. Sci. B.

[54] Toda M., Walder F., van der Heijden M.G.A. (2023). Organic Management and Soil Health Promote Nutrient Use Efficiency. J. Sustain. Agric. Environ..

[55] Jia Y., Hu Z., Ba Y., Qi W. (2021). Application of Biochar-Coated Urea Controlled Loss of Fertilizer Nitrogen and Increased Nitrogen Use Efficiency. Chem. Biol. Technol. Agric..

[56] Tripti, Kumar A., Usmani Z., Kumar V., Anshumali (2017). Biochar and Flyash Inoculated with Plant Growth Promoting Rhizobacteria Act as Potential Biofertilizer for Luxuriant Growth and Yield of Tomato Plant. J. Environ. Manag..

[57] Murugan P., Ong S.Y., Hashim R., Kosugi A., Arai T., Sudesh K. (2020). Development and Evaluation of Controlled Release Fertilizer Using P(3HB-*Co*-3HHx) on Oil Palm Plants (Nursery Stage) and Soil Microbes. Biocatal. Agric. Biotechnol..

[58] Iftime M.M., Ailiesei G.L., Ungureanu E., Marin L. (2019). Designing Chitosan Based Eco-Friendly Multifunctional Soil Conditioner Systems with Urea Controlled Release and Water Retention. Carbohydr. Polym..

[59] Lu H., Tian H., Liu Z., Zhang M., Zhao C., Guo Y., Guan R., Chen Q., Yu X., Wang H. (2019). Polyolefin Wax Modification Improved Characteristics of Nutrient Release from Biopolymer-Coated Phosphorus Fertilizers. ACS Omega.

[60] Kandaiah R., Ravindran A., Panneerselvan L., Manivannan A.C., Kulanthaisamy M., Sobhani Z., Bhagwat-Russell G., Palanisami T. (2024). A Comprehensive Analysis and Risk Evaluation of Microplastics Contamination in Australian Commercial Plant Growth Substrates: Unveiling the Invisible Threat. J. Hazard. Mater..

[61] Quynh H.T., Kazuto S. (2018). Title “Organic Fertilizers” in Vietnam’s Markets: Nutrient Composition and Efficacy of Their Application. Sustainability.

[62] Tian H., Chen Q., Liu Z., Zhang M., Abolfathi S., Tanentzap A.J. (2026). Long-Term Localization Experiments Reveal Aging Degradation Mechanisms of Biobased and Petroleum-Based Polyurethanes in Natural Environments: Degradation Characteristics, Product Assessment and Degradation Cycle Prediction. Environ. Pollut..

[63] Jumakir J., Waluyo W., Sutrisna N., Pardal S.J., Jauhari S., Suprihatin A., Budiarti S.W., Setiyawan A., Cahya M., Putra N.R. (2026). Coating-Engineered Controlled-Release Urea Fertilizers: A Systematic Review of Release Mechanisms, Performance Trade-Offs, and Environmental Implications. J. Appl. Polym. Sci..

[64] Brunelle T., Dumas P., Souty F., Dorin B., Nadaud F. (2015). Evaluating the Impact of Rising Fertilizer Prices on Crop Yields. Agric. Econ..

